# Diverse modes of binding in structures of *Leishmania major* *N*-myristoyltransferase with selective inhibitors

**DOI:** 10.1107/S2052252514013001

**Published:** 2014-06-17

**Authors:** James A. Brannigan, Shirley M. Roberts, Andrew S. Bell, Jennie A. Hutton, Michael R. Hodgkinson, Edward W. Tate, Robin J. Leatherbarrow, Deborah F. Smith, Anthony J. Wilkinson

**Affiliations:** a Structural Biology Laboratory, Department of Chemistry, University of York, York YO10 5DD, England; b Department of Chemistry, Imperial College London, South Kensington Campus, London SW7 2AZ, England; c Centre for Immunology and Infection, Department of Biology, University of York, York YO10 5DD, England

**Keywords:** *N*-myristoyltransferase, inhibitor, ligand binding, *Leishmania*, drug discovery

## Abstract

The leishmaniases are a spectrum of global diseases of poverty associated with immune dysfunction and are the cause of high morbidity. Despite the long history of these diseases, no effective vaccine is available and the currently used drugs are variously compromised by moderate efficacy, complex side effects and the emergence of resistance. It is therefore widely accepted that new therapies are needed. *N*-Myristoyltransferase (NMT) has been validated pre-clinically as a target for the treatment of fungal and parasitic infections. In a previously reported high-throughput screening program, a number of hit compounds with activity against NMT from *Leishmania donovani* have been identified. Here, high-resolution crystal structures of representative compounds from four hit series in ternary complexes with myristoyl-CoA and NMT from the closely related *L. major* are reported. The structures reveal that the inhibitors associate with the peptide-binding groove at a site adjacent to the bound myristoyl-CoA and the catalytic α-carboxylate of Leu421. Each inhibitor makes extensive apolar contacts as well as a small number of polar contacts with the protein. Remarkably, the compounds exploit different features of the peptide-binding groove and collectively occupy a substantial volume of this pocket, suggesting that there is potential for the design of chimaeric inhibitors with significantly enhanced binding. Despite the high conservation of the active sites of the parasite and human NMTs, the inhibitors act selectively over the host enzyme. The role of conformational flexibility in the side chain of Tyr217 in conferring selectivity is discussed.

## Abstract 2

Crystal structures of *N*-myristoyltransferase with four distinct *Leishmania*-selective small-molecule inhibitors identify key binding-site residues and suggest strategies to design compounds with increased affinity.

## 1. Introduction   

The leishmaniases, caused by species of the kinetoplastid parasite *Leishmania*, are a spectrum of diseases associated with immune dysfunction, with ∼350 million people at risk in 98 countries where these diseases are endemic (Alvar *et al.*, 2012 ▶). Clinical symptoms range from the disfiguring skin lesions of cutaneous leishmaniasis (CL) to the often fatal visceral leishmaniasis (VL) characterized by prolonged fever, enlarged spleen and liver and progressive anaemia. These symptoms are exacerbated in children and the immuno­compromised, such as those diagnosed as human immuno­deficiency virus (HIV) positive, and HIV–VL co-infection is an increasing problem (Alvar *et al.*, 2008 ▶).

Almost all clinically symptomatic VL patients die within months if untreated. There are currently no anti-leishmanial vaccines licensed for use in humans. The principal drugs used to treat visceral leishmaniasis have been pentavalent antimonials, but these compounds have toxic side effects and their effectiveness is threatened by the emergence of drug resistance, especially in the Indian subcontinent. The recognized alternatives, miltefosine, amphotericin B and paromomycin, suffer from various drawbacks including lack of an oral formulation, prolonged treatment times, high costs of treatment and toxicity. As a result, the development of new therapies for treating leishmaniasis is an international priority. Previous studies have identified myristoyl-CoA:protein *N*-myristoyltransferase (NMT) as a promising candidate for drug development against pathogenic protozoan parasites (Price *et al.*, 2003 ▶; Panethymitaki *et al.*, 2006 ▶; Bowyer *et al.*, 2007 ▶, 2008 ▶; Brannigan *et al.*, 2010 ▶; Frearson *et al.*, 2010 ▶).

**Figure 1:**
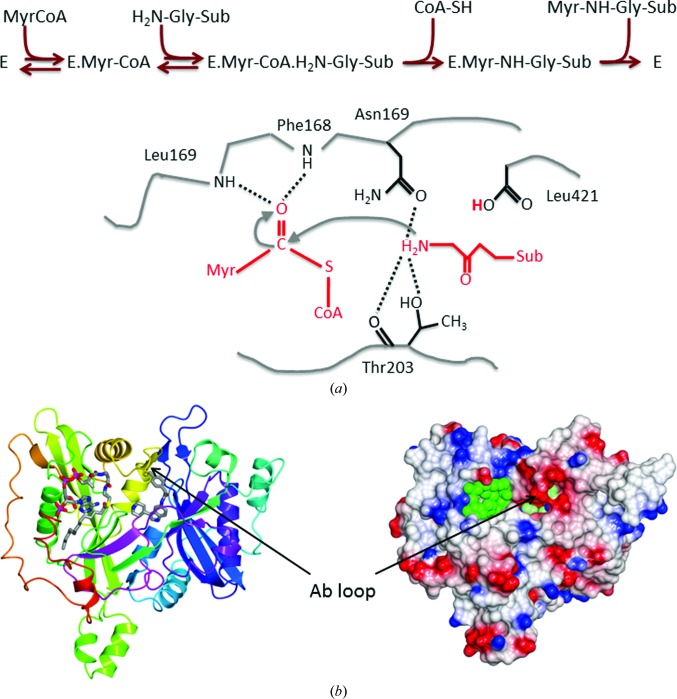
The reaction catalysed by *N*-myristoyltransferase. (*a*) Top, scheme showing the ordered binding of myristoyl-CoA (MyrCoA) and the substrate protein followed by the ordered release of CoASH and the myristoylated product. Bottom, schematic of a step in the reaction mechanism showing the active site following binding of substrates and abstraction by the α-carboxylate of the C-terminal residue Leu421 of a proton from the α-amino group of Gly1 of the substrate protein. Thr303 and Asn169 form polar interactions with the amino group which attacks the carbonyl C atom of MyrCoA. The reaction intermediate is stabilized by interactions with an oxyanion hole formed by the amides of Leu167 and Phe168. This mechanism is adapted from Farazi *et al.* (2001 ▶). (*b*) Ribbon (left) and electrostatic surface (right) representations of LmNMT with bound MyrCoA and compound 7AH. The ligands bind in an extended cleft running across the molecule that is partially covered by the Ab loop. In the left-hand image the ligands are displayed as cylinders and coloured by atom type; in the right-hand image MyrCoA and 7AH are shown as green and light green spheres, respectively.

In eukaryotic cells, NMT catalyses the transfer of the 14-carbon saturated fatty acid myristate from myristoyl-CoA (MyrCoA) to the amino-terminal glycine of a subset of proteins. This predominately co-translational modification contributes to the targeting of substrate proteins to membrane locations as well as facilitating protein–protein interactions (Resh *et al.*, 2012 ▶). *N*-Myristoylation by NMT proceeds *via* an ordered bi-bi reaction mechanism (Fig. 1 ▶ *a*): binding of MyrCoA generates small conformational changes that enable docking of the substrate protein and deprotonation of its α-amino group by the α-carboxylate of the C-terminal residue acting as a base (Rudnick *et al.*, 1991 ▶; Bhatnagar *et al.*, 1994 ▶). The myristate group is then transferred to the N-terminal glycine of the substrate in a nucleophilic addition–elimination reaction with the formation of an amide bond (Fig. 1 ▶ *a*). There follows stepwise release of first the free CoA and then the *N*-myristoylated protein (Rudnick *et al.*, 1991 ▶; Bhatnagar *et al.*, 1999 ▶). NMTs have been well characterized in *Saccharo­myces cerevisiae* (Duronio *et al.*, 1989 ▶) and human cells (Ducker *et al.*, 2005 ▶) and are essential for viability in pathogenic fungi (Lodge *et al.*, 1994 ▶).

Comparative sequence and biochemical analyses demonstrated high conservation of the MyrCoA binding sites in the two human isoforms of the enzyme, HsNMT1 and HsNMT2, and in the fungal NMTs, but divergent peptide-binding specificities (Johnson *et al.*, 1994 ▶). This led to the development of peptide-based and peptidomimetic inhibitors that showed selectivity against the NMT from *Candida albicans* relative to human NMT (Lodge *et al.*, 1997 ▶, 1998 ▶). As a consequence, NMT was the target of antifungal drug-development programmes in the pharmaceutical industry, with the focus on selective inhibitors acting at the peptide-binding pocket. In the preliminary stages of these programs, high selectivity and specificity were achieved around benzothiazole (Pfizer, unpublished work) and benzofuran (Roche; Masubuchi *et al.*, 2001 ▶, 2003 ▶) scaffolds. However, the best leads proved to be specific for *C. albicans* and unlikely to give rise to the types of broad-spectrum drugs (ideally also active against *Aspergillus* and *Cryptococcus* spp.) that would enable them to compete with current antifungal drugs. Cross-species activity is not essential in drug development for parasitic infections, which are readily diagnosed according to clinical, molecular and epidemiological indicators. Although there is no conclusive evidence for toxic effects arising from inhibition of either human NMT, selectivity for the appropriate parasitic NMT is highly desirable.

These considerations suggested NMT as a suitable target for developing chemotherapeutics against infectious parasites (Tate *et al.*, 2014 ▶) to treat diseases such as malaria (caused by *Plasmodium* spp.), leishmaniasis (*Leishmania* spp.) or African sleeping sickness (*Trypanosoma brucei*). To substantiate this hypothesis, the NMTs of *L. major* and *L. donovani* (which cause CL and VL, respectively), *P. falciparum* and *T. brucei* (Price *et al.*, 2003 ▶; Panethymitaki *et al.*, 2006 ▶; Bowyer *et al.*, 2007 ▶, 2008 ▶; Brannigan *et al.*, 2010 ▶) were characterized and shown to be essential for the viability of these species using targeted gene disruption, RNAi techniques and chemical biology approaches (Price *et al.*, 2003 ▶, 2010 ▶; Wright *et al.*, 2014 ▶). The validity of NMT as a drug target was demonstrated by the use of high-throughput screening to produce a small-molecule inhibitor of *T. brucei* NMT that killed bloodstream parasites *in vivo* with high sensitivity and specificity (Frearson *et al.*, 2010 ▶). Most recently, inhibitors of NMT from *Plasmodium* have been developed (Goncalves, Brannigan, Whalley *et al.*, 2012 ▶; Yu *et al.*, 2012 ▶; Rackham *et al.*, 2013 ▶, 2014 ▶) and were shown to disrupt the formation of critical subcellular structures, leading to rapid parasite cell death (Wright *et al.*, 2014 ▶).

A complementary high-throughput screen of over 150 000 compounds from the Pfizer Global Diverse Representative Set against protozoan parasite NMT proteins (Bell *et al.*, 2012 ▶) identified a number of submicromolar inhibitors of *L. donovani* NMT which also displayed selectivity over the host (human) enzyme. Since the published *in vivo* active inhibitors of *T. brucei* NMT are reported to have single-digit nanomolar activity in enzyme and cellular assays, we envisaged that a further 100-fold to 1000-fold improvement in enzyme affinity would be required to produce a useful clinical candidate using a structure-guided approach.

We have resynthesized selected samples of the high-throughput screen hits (or a close analogue in one case) and now report the binding modes of four distinct *Leishmania*-selective small-molecule inhibitors in crystal structures of ternary complexes with *L. major* NMT and MyrCoA co-substrate. Analysis of the crystal structures has identified key binding-site residues and strategies to modify the inhibitors to achieve the desired increase in enzyme affinity and selectivity over the human NMTs.

## 2. Materials and methods   

### 2.1. Protein preparation and crystallization   

Protein expression and purification was essentially as described for LdNMT (Brannigan *et al.*, 2010 ▶) using clone LmNMT_SGC:B1 that encodes an N-terminal histidine tag and a cleavage site for TEV protease (MHHHHHHSSGRE­NLYFQG) followed by residues 5–421 of LmNMT (Frearson *et al.*, 2010 ▶). Protein at 10 mg ml^−1^ was incubated at 4°C overnight with a 1/20th volume of cofactor MyrCoA (10 m*M* in 50% DMSO) and crystallized by vapour diffusion using a mother liquor consisting of 30% PEG 1500, 0.2 *M* NaCl, 0.1 *M* sodium cacodylate pH 5.5. For co-crystallization of 7AH, ligand at a final concentration of 1 m*M* was incubated with LmNMT and MyrCoA as above (32% PEG 1500, 0.2 *M* NaCl, 0.1 *M* sodium cacodylate pH 5.6). For the other ligands, crystal-soaking experiments proved to be more reliable and convenient. Ligand compounds (25 m*M* stocks in 50% DMSO) were added to a stabilization solution (33% PEG 1500, 0.22 *M* NaCl, 0.11 *M* sodium cacodylate pH 5.5) to give a final ligand concentration of 2.5 m*M*. Ligand solution was used to replace liquid in crystallization drops containing LmNMT–MyrCoA crystals by careful pipetting, repeated three times to completely wash away the original drop solution and left to soak for 20 h.

### 2.2. Data collection and refinement   

X-ray diffraction data were collected on synchrotron beamlines at the Diamond Light Source and were processed using *XDS* (Kabsch, 2010 ▶) and *SCALA* (Evans, 2006 ▶) implemented within *xia*2 (Winter, 2010 ▶). Data-collection and refinement statistics are summarized in Table 2. The protein coordinates from PDB entry 3h5z (Frearson *et al.*, 2010 ▶) were used directly for refinement using the maximum-likelihood methods implemented in *REFMAC* (Murshudov *et al.*, 2011 ▶). Cycles of refinement using anisotropic temperature factors were interspersed with model building and adjustment using *Coot* (Emsley *et al.*, 2010 ▶). The complete chain can be traced for the protein, with the exception of the N-terminal residues preceding Ala11 (numbering as in the full-length native LmNMT protein). These residues are not defined in the electron-density maps and they are assumed to be disordered. The final refined protein structure model displays good geometry, with 97% of the residues in the preferred region of the Ramachandran plot and only 0.3% (corresponding to amino-acid residue His347) as outliers. The coordinates and structure-factor files have been deposited in the Protein Data Bank under accession codes 4cgp (LmNMT–MyrCoA), 4cgo (LmNMT–MyrCoA–6KV), 4cgn (LmNMT–MyrCoA–7AH), 4cgl (LmNMT–MyrCoA–A6K) and 4cgm (LmNMT–MyrCoA–CWZ).

## 3. Results and discussion   

A high-throughput screen against *L. donovani* NMT (Bell *et al.*, 2012 ▶) identified a number of compound classes with submicromolar inhibition of the parasite NMT and selectivity against human NMT. Four chemically distinct scaffolds (thieno­pyrimidine, piperidinylindole, aminoacylpyrrolidine and biphenyl) were disclosed to enable the generation of drug-like lead compounds (Table 1 ▶). In following up on this publication, our initial objective was a resynthesis of the hits from the high-throughput screen to validate their activity in NMT assays and then to support profiling in secondary assays, including cell-based assays and structural biology studies. In two cases (piperidinylindole and biphenyl derivatives), the targets were obtained in a straightforward manner. The aminoacylpyrrolidine derivative has three chiral centres. As the absolute stereochemistry of the pyrrolidine moiety was uncertain, we synthesized this hit as a mixture of diastereomers, envisioning that the most active isomer would be identified in the co-crystal structure. Finally, in an attempt to control the ultimate cost of goods, we opted to synthesize the desmethyl analogue of the thienopyrimidine (Table 1 ▶ and Supporting Information). The details of the synthesis of these compounds will be reported elsewhere.

The four newly synthesized compounds were screened in enzyme assays against the same NMT proteins as used in the Pfizer screen, but using an improved assay (Goncalves, Brannigan, Thinon *et al.*, 2012 ▶) which avoids the use of radioactive substrates. In addition, the compounds were tested against *L. major* NMT to determine their affinity for the enzyme used for structure determination (Table 1 ▶). The activity of the newly resynthesized compounds was consistent with the originally reported data (Bell *et al.*, 2012 ▶).

To underpin investigations into structure–activity relationships, we sought to prepare crystals of ternary complexes of *L. donovani* NMT (LdNMT) with MyrCoA and each of the inhibitors listed in Table 1 ▶. However, we were unsuccessful in soaking inhibitors into crystals of LdNMT, a failure that we attribute to the lattice interactions in the crystals, which restrict access to the ligand-binding groove (Brannigan *et al.*, 2010 ▶). We were similarly unsuccessful in co-crystallization approaches to ternary-complex crystals. We therefore turned to *L. major* NMT (LmNMT), which has been shown to give crystals which are amenable to the introduction of ligands by crystal soaking (Frearson *et al.*, 2010 ▶). LmNMT and LdNMT have highly similar sequences, differing at just 11 of 421 positions (97.5% identical), and their structures are closely superimposable. Thus, we were able to prepare crystals of LmNMT in complex with MyrCoA and each of the four inhibitor compounds. The crystals diffracted to high resolution and data sets extending to 1.3–1.7 Å spacing were collected (Table 2 ▶).

### 3.1. Overall structure of the LmNMT–MyrCoA–inhibitor complexes   

The crystal structures reported here are isomorphous with those reported previously for LmNMT in complexes with bound MyrCoA (PDB entry 3h5z) and MyrCoA plus inhibitor DDD85646 (PDB entry 2wsa), respectively (Frearson *et al.*, 2010 ▶). A representative structure, that of LmNMT in complex with MyrCoA and the piperidinylindole 7AH, is shown in Fig. 1 ▶(*b*). The structure of LmNMT, like those of other NMTs (Weston *et al.*, 1998 ▶; Bhatnagar *et al.*, 1999 ▶), consists of a twisted central β-sheet onto which helices are packed so as to form an extended and curved substrate-binding groove that runs across two protein lobes. The MyrCoA co-substrate binds to the amino-terminal lobe with the alkane moiety buried in a deep hydrophobic pocket and the thioester group in close proximity to the α-carboxylate of the C-terminal residue Leu421, which plays a catalytic role. The CoA moiety adopts a compact structure, with the adenine ring surrounded by the pantetheine and fatty-acyl species. Compound 7AH binds to the C-terminal lobe that has been shown in the NMTs from *S. cerevisiae* (Bhatnagar *et al.*, 1998 ▶; Farazi *et al.*, 2001 ▶) and *C. albicans* (Sogabe *et al.*, 2002 ▶) to form the binding site for peptide substrates or peptidomimetic inhibitors (Fig. 1 ▶ *b*).

The five LmNMT structures presented here are closely similar, with pairwise values for the root-mean-square deviation (r.m.s.d.) in C^α^ coordinate positions in the range 0.2–0.6 Å (Supplementary Fig. S1*a*). The MyrCoA binding sites are closely similar and the mode of co-substrate binding is identical, as shown in Supplementary Fig. S1(*b*). The most notable structural changes accompanying binding of the inhibitors take place in the acidic loop containing residues Glu82-Asp-Asp-Asp85 (the Ab loop), which has been termed a lid that closes over the active site upon substrate binding (Fig. 1 ▶), and the glycine-rich segment Gly393-Ala-Gly-Asp-Gly397 situated on the lower surface of the binding site. Comparison of the binary complexes of LmNMT with MyrCoA and of LdNMT with a nonhydrolysable analogue of the co-substrate (PDB entry 2wuu; Brannigan *et al.*, 2010 ▶) gives an r.m.s.d. value of 1.0 Å for 402 equivalent C^α^ positions.

### 3.2. Binding of the thienopyrimidine compound 6KV   

**Figure 2:**
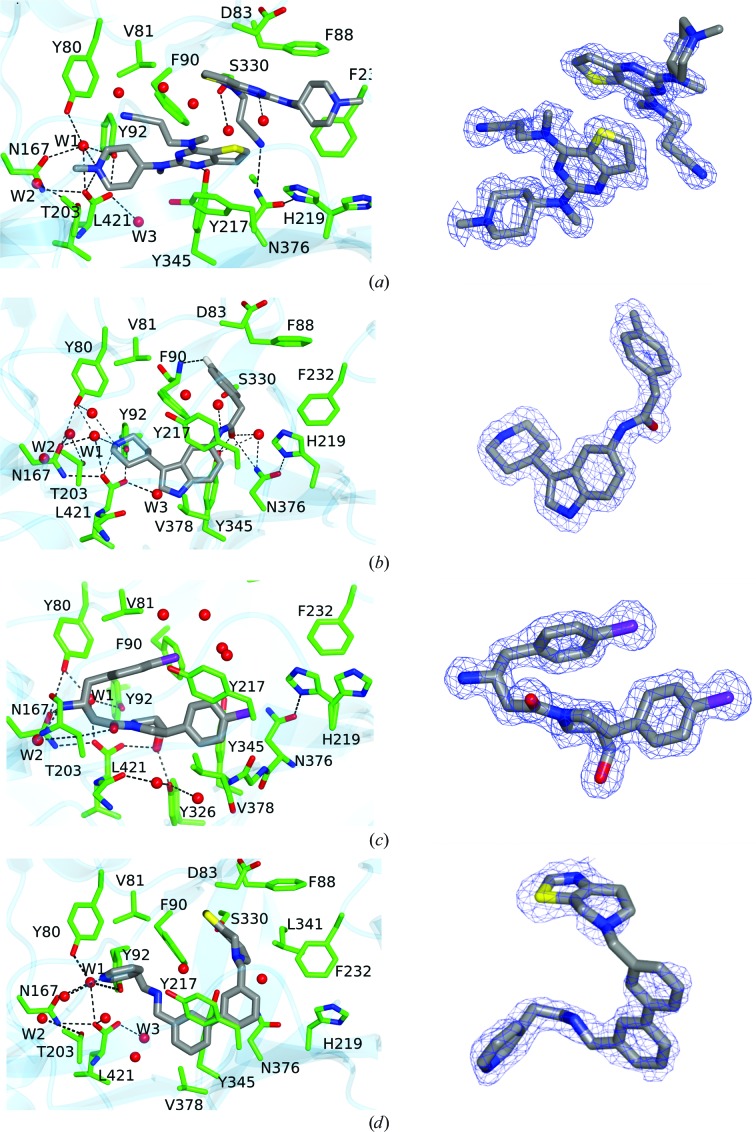
The left-hand panels show the binding site for each ligand: (*a*) the thienopyrimidine 6KV, (*b*) the piperidinylindole 7AH, (*c*) the aminoacylpyrrolidine A6K and (*d*) the biphenyl derivative CWZ. The structures are coloured by atom; carbon, green for protein and grey for ligand; oxygen, red; nitrogen, blue; sulfur, yellow; fluorine, silver; chlorine, mauve. Selected water molecules within 3.5 Å of the ligands are also shown. For clarity, the co-substrate MyrCoA is not shown. Stereoviews (including the co-substrate) of the binding site are presented in Supplementary Fig. S2. The right-hand panel shows the final refined electron-density map associated with bound ligand (2*mF*_o_ − *DF*_c_) contoured at a level of 1σ.

**Figure 3:**
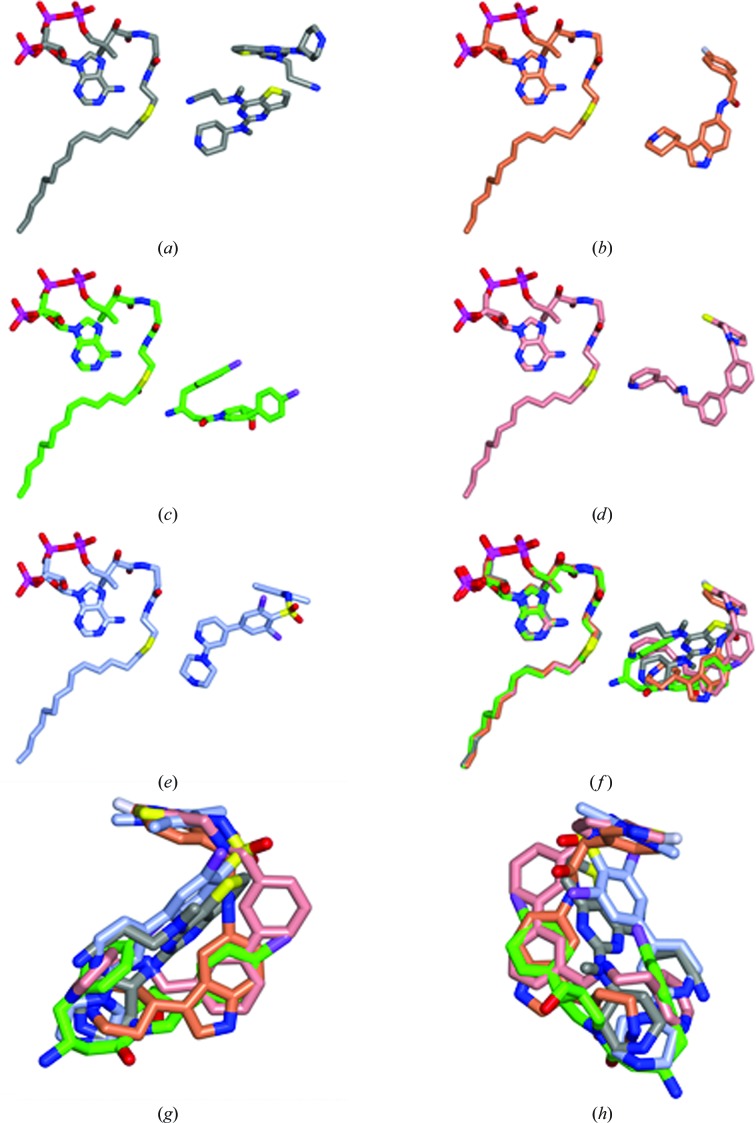
Binding of ligands and MyrCoA cofactor. Molecules are in cylinder representation in all panels. The atoms are coloured by element (oxygen, red; nitrogen, blue; sulfur, yellow; phosphorus, magenta; fluorine, silver; chlorine, mauve) with the C atoms coloured by inhibitor: (*a*) 6KV, grey; (*b*) 7AH, coral; (*c*) A6K, green; (*d*) CWZ, pink; (*e*) 646 (PDB entry 2wsa), ice blue. In (*f*) the MyrCoA and the inhibitors in (*a*)–(*d*) are overlaid. (*g*, *h*) Two views of the inhibitor ligands overlaid. For clarity, only the proximal 6KV molecule is shown in the overlaps.

This inhibitor is a close analogue of the original high-throughput screen hit, the only difference being the deletion of the methyl substituent on the thienopyrimidine ring (Table 1 ▶). Its biological profile is similar to the original hit compound, with an IC_50_ value for LdNMT of 0.25 µ*M* and modest selectivity (approximately tenfold) against human NMT isoform 1 (HsNMT1). Unexpectedly, two molecules of compound 6KV occupy the peptide-binding groove of LmNMT (Fig. 2 ▶ *a*, Supplementary Fig. S2*a*). These will be referred to as the proximal (P) and distal (D) ligands based on their positions relative to the MyrCoA ligand (Fig. 3 ▶ *a*, Table 3 ▶).

The proximal ligand is expected to be the higher affinity binding ligand based on its fit to the electron-density maps (Fig. 2 ▶ *a*, Supplementary Fig. S3*a*) and its lower mean atomic temperature (*B*) factor. As is evident, the thienopyrimidine rings pack together. The two ligands bury 390 and 400 Å^2^, respectively, of their surface area through contacts with the protein and a further 80 Å^2^ through contacts with each other (Table 3 ▶). The two molecules are related to one another by a rotation about an approximately vertical axis, as viewed in Fig. 2 ▶(*a*), followed by a translation in the vertical direction. The piperidine ring in the distal inhibitor molecule is poorly defined in the electron-density maps. The mean temperature factor of the atoms of the *N*-methylpiperidine moiety is 25 Å^2^ higher than the mean value for the other atoms of the distal ligand. This suggests that there is rotation about the N4—C11 bond and that the distal piperidine ring adopts an ensemble of conformations. The presence of the ligand(s) induces ordering of a loop implicated in substrate binding which contains the conserved residue Gly397. Val81, Phe90 and His219 (which is modelled in two conformations) form significant interactions with both ligand molecules. Asp83, Phe88, Phe232 and Asp396 are prominent in binding the D ligand molecule, with Gly205 and Tyr217 forming significant interactions exclusively with the P ligand.

There are few obvious polar inter­actions between the protein and either inhibitor molecule. There is a direct hydrogen bond between the phenolic hydroxyl of Tyr345 (2.7 Å) and the ring N atom N4 of the pyrimidine ring. The *N*-methylpiperidine ring is protonated so it will form an ionic interaction with the carboxylate of Leu421, from which it is separated by 3.2 Å. The same N atom forms a hydrogen bond (2.9 Å) to a well ordered water molecule (W1), which may additionally form polar inter­actions with the C-terminal carboxylate and the side chains of residues Tyr80, Tyr92 and Asn167. The pendant nitrile group in the proximal ligand is oriented towards the MyrCoA (nitrile N to cofactor S distance of 4.2 Å) and is surrounded by Val81, Ala204, Gly205 and a number of solvent molecules. For the distal ligand, this moiety is projecting into a pocket circumscribed by Phe90, Phe232, Ser330, Leu341, Ala343, Tyr345 and Val374, with the potential for a hydrogen bond between the N atom of the nitrile and the side-chain amino group of Asn376 (3.2 Å).

### 3.3. Binding of the piperidinylindole compound 7AH   

Compound 7AH inhibits LdNMT with an IC_50_ value of 0.3 µ*M* with excellent (∼200-fold) selectivity against HsNMT1 (Table 1 ▶). When bound to LmNMT, 7AH effectively wraps around the side chain of Phe90, with its phenyl and piperidine rings packing onto opposite faces of the aromatic ring of the side chain; meanwhile, the amide linker between the indole and fluoro­phenyl species packs against the edge of the Phe90 ring (Fig. 2 ▶ *b*, Supplementary Fig. S2*b*). 510 Å^2^ of its 570 Å^2^ of surface area is buried by interaction with the protein. The faces of the indole ring itself pack against the edges of the aromatic rings of the side chains of Tyr217 and Tyr345. In this structure, there is a noticeable ordering of the Ab loop containing the acidic segment Glu82-Asp-Asp-Asp85 and there is the possibility of a polar interaction with fluorine. Since this halogen atom rarely acts as a hydrogen-bond acceptor, we postulate a dipole–dipole interaction with a backbone amide group.

The piperidine ring forms an ion pair (2.8 Å) with the α-carboxylate of Leu421 and is additionally solvated by two water molecules which include W1 and form a local network of polar interactions extending to residues Tyr80, Tyr92, Asn167 and Thr203. Elsewhere, the carbonyl of the amide of the ligand forms hydrogen bonds to the side-chain hydroxyl of Tyr345 (2.7 Å) and the amide amino group of Asn376 (3.2 Å), the amide carbonyl of which forms a strong hydrogen bond to N^δ^ of His219, which is well ordered in this complex. This local network of hydrogen bonding is completed by a water molecule which forms hydrogen bonds to the ligand carbonyl and the side chains of residues Tyr345 and Asn376.

### 3.4. Binding of the amino­acylpyrrolidine compound A6K   

This ligand was identified through analogue screening of the primary high-throughput screening hit and has the highest IC_50_ value (0.08 µ*M* for LdNMT) among the four inhibitors presented here. Its IC_50_ against the human enzyme is 5 µ*M*, giving a selectivity factor of ∼80 (Table 1 ▶). Compound A6K is seen bound as a single diastereomer (*SRR*) and strikingly has a compact structure when bound to LmNMT (Fig. 2 ▶ *c*, Supplementary Fig. S2*c*). Adoption of this type of conformation has been referred to as hydrophobic collapse (Wiley & Rich, 1993 ▶). The first chloro­phenyl group packs between Tyr217 and Tyr345 and above Val378, with the plane of its ring perpendicular to the plane of the pyrrolidine ring. The second of the two chlorophenyl rings is folded back over the pyrrolidine ring, with which it lies in an approximately parallel plane. It makes extensive apolar interactions with Val81 and Phe90.

The exocyclic hydroxymethyl group displaces a water molecule (W3), the position of which is conserved in the other ligand complexes, and makes two strong interactions with the hydroxyl group of Tyr326 (2.6 Å) and an O atom of the α-carboxylate of Leu421 (2.6 Å) as part of a local network of polar interactions that include the carbonyl O atom of Met420. The folding of the inhibitor projects the primary amino group in the direction of the MyrCoA co-substrate. Thr203 makes bridging contacts to this primary amine group as well as to the carbonyl O atom of the ligand through hydrogen bonds formed to its main-chain carbonyl and side-chain hydroxyl groups, respectively. The primary amine of the inhibitor forms additional polar interactions with the side-chain amide carbonyl of Asn167 and a water molecule (W2); the latter also forms hydrogen bonds to the main-chain amide and carbonyl groups of Thr203.

The chloro substituents of the two aromatic rings project away from the protein core. One Cl atom (Cl1) is surrounded by four water molecules, two of which are within 3.3 Å. It is 3.9 Å from the Tyr217 hydroxyl, and could contribute a relatively weak binding interaction, given the potential for organic chlorines to interact with protein Lewis bases *via* halogen bonding (Sirimulla *et al.*, 2013 ▶).

### 3.5. Binding of the biphenyl-derivative compound CWZ   

This inhibitor has an IC_50_ value of 0.9 µ*M* for LdNMT and 45 µ*M* for HsNMT1, giving a selectivity factor of 50. The relatively weak binding of this compound is reflected in the initial electron-density OMIT maps (Supplementary Fig. S3). Relative to the other three inhibitors described here, it also has a low ligand efficiency value (Hopkins *et al.*, 2014 ▶) of 0.29 (Table 1 ▶). The binding of this ligand has the effect of ordering the acidic Glu82–Asp85 region of the Ab loop and there is a single orientation of residue His219, which has lost its interaction with Asn376.

This ligand makes extensive and largely apolar interactions with the protein, with 630 of its 700 Å^2^ of accessible surface area buried by interactions with protein residues. In a similar manner to compound 7AH, compound CWZ wraps around the side-chain aromatic group of Phe90, while the biphenyl species packs between the side chains of Tyr217 and Tyr345 (Fig. 2 ▶ *d*, Supplementary Fig. S2*d*). There is a dearth of polar protein–inhibitor interactions. The only obvious strong hydrogen bond is formed between the N atom of the thiazole moiety and the hydroxyl of Ser330, where the contact distance is 2.7 Å. At the other end of the molecule, there are two possible orientations of the pyridine ring arising from rotation about the C1—C2 bond. In the orientation shown, the pyridine N atom is situated 3.5 Å from a water molecule and 3.7 Å from the S atom of the MyrCoA co-substrate; in the other orientation it would be 3.7 Å from the phenolic hydroxyl of Tyr345.

### 3.6. Comparison of binding with the inhibitor DDD85646   

DDD85646 (646) is a potent inhibitor of NMT from *T. brucei* (IC_50_ = 2 n*M*) that was evolved from a compound discovered in a high-throughput screen (Frearson *et al.*, 2010 ▶). This compound has low selectivity, inhibiting HsNMT1 with an IC_50_ of 12 n*M* and LdNMT with an IC_50_ value of below 6 n*M*. As shown in Fig. 3 ▶(*e*), Supplementary Fig. S2(*e*) and Table 3 ▶, when bound to LmNMT compound 646 occupies the same pocket as the inhibitors described above, contacting essentially the same set of protein residues. It binds in a reasonably extended conformation, with three of its ring elements being close to coplanar but with the pyrazole ring projecting in a perpendicular direction, induced by the sulfonamide linker. At the extremes of the molecule, the piperazine N atom forms an ion-pairing interaction with the α-carboxylate of Leu421, while the N atom of the pyrazole forms a hydrogen bond to the hydroxyl of Ser330. Of the inhibitors discussed above, CWZ alone forms a hydrogen bond to Ser330. However, CWZ is also alone in failing to form a direct interaction with Leu421. Thus, the capacity to span the pocket and develop polar interactions with both Ser330 and Leu421 in the leishmanial NMTs, built into DDD85646 during an extended medicinal chemistry campaign, probably accounts for its much higher potency relative to the high-throughput screen hits described here. From the superposition shown in Fig. 3 ▶, it is apparent that the three contiguous rings of 646 overlap most closely with the proximal-pocket bound 6KV, with the fourth pyrazole ring overlaying the fluorophenyl ring of 7AH and the thiazolopiperidine ring of CWZ. In contrast to the latter two ligands, 646 navigates a more direct route from the vicinity of the protein C-terminus to the distal site.

### 3.7. Comparison of the inhibitor-binding sites and the basis of selectivity   

**Figure 4:**
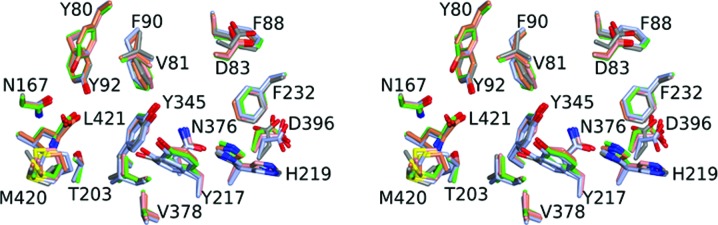
Comparison of the inhibitor-binding sites. The stereo image shows the residues (labelled) that circumscribe the inhibitor-binding site in LmNMT. The colouring is by atom type, with oxygen in red, nitrogen in blue and sulfur in yellow. C atoms are coloured according to the structure as follows. Those in the binary complex of LmNMT with MyrCoA are shown in ice blue and those in the ternary complexes of LmNMT–MyrCoA with the inhibitors 6KV, 7AH, A6K and CWZ are shown in grey, coral, green and pink, respectively. The structures were superposed using the secondary-structure matching routines implemented in *CCP*4*mg* (McNicholas *et al.*, 2011 ▶).

The inhibitor-binding sites of the four ternary complexes described above together with that in the LmNMT–MyrCoA binary complex are compared in Fig. 4 ▶. A number of residues are modelled in two conformations consistent with the electron-density maps. His219 is especially interesting in its conformational flexibility. In the binary complex and in the ternary complexes with the ligands 6KV and A6K, two conformations (inward and outward) are evident. In the complex with 7AH, the inward (*m*, gauche−) conformation alone is apparent, in contrast to the complex with CWZ where the outward (*p*, gauche+) conformation alone is observed. In the 7AH complex, the inward orientation of His219 is favoured by the hydrogen-bonding network extending from the ligand *via* the Asn376 side-chain amide to the imidazole side chain. In contrast, in the CWZ complex the inward conformation is sterically hindered by the biphenyl moiety. His219 has been shown to be an important residue in yeast NMT since a mutant with a substitution at this position is not viable (Farazi *et al.*, 2001 ▶). In complex with the peptide GLYASKLA, His219 interacts with Ser5, which is a strongly preferred residue at this position in substrates of NMT.

Also apparent from Fig. 4 ▶ are distinct conformations of residue Tyr217. An inwardly directed conformer is observed in the ternary complex with 6KV, while the other outwardly directed conformer is observed in the complexes with 7AH, A6K and CWZ. In the binary complex with MyrCoA both conformers are present. Tyr217 is a key residue in the binding of all four inhibitors, contributing 34–58 Å^2^ of interfacial surface.

The selectivity of the inhibitors for the NMTs from *Leishmania* relative to those from human cannot be explained straightforwardly by mapping the residues contacting the inhibitors in the structures onto the aligned sequences of the proteins. On the contrary, all of the amino-acid side chains that make significant interactions with the inhibitors in LdNMT/LmNMT, defined either by a sizable contribution to the ligand interface or their participation in polar interactions with the latter (Table 3 ▶), are conserved in human NMT.

**Figure 5:**
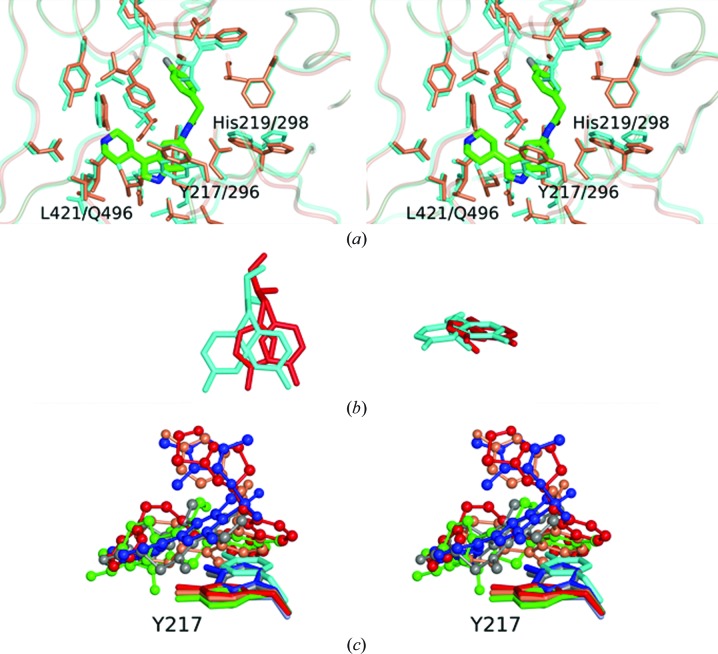
Conformational flexibility of residue Tyr217. (*a*) Stereo overlay of the LmNMT–MyrCoA structure (coral) with HsNMT–MyrCoA (PDB entry 3iu1, cyan). The position of binding for the inhibitor 7AH is shown for reference. (*b*) Preferred positions of the LmNMT Tyr217 (red) and HsNMT Tyr296 (cyan) conformers. (*c*) Clustering of the various inhibitors. No inhibitor (HsNMT), cyan; DDD85646, blue; 6KV, grey; 7AH, coral; A6K, green; CWZ, red. For clarity, only the proximal 6KV molecule is shown. An inward orientation of the Tyr217 side chain prevails in the complexes with DDD85646 and 6KV, while outward orientations are observed in the complexes with 7AH, A6K and CWZ.

To explore the origins of selectivity in more detail, we compared the structure of the LmNMT complex with MyrCoA with that of the binary complex of HsNMT with MyrCoA (PDB entry 3iu1; Structural Genomics Consortium, unpublished work). This comparison confirms the similarity of the inhibitor-binding pockets in the two enzymes and the close superposition of corresponding residues (Fig. 5 ▶ *a*). The most obvious differences occur in the Ab loop, which is ‘more closed’ in the human NMT. The comparison suggests Tyr217 (Tyr296 in HsNMT) as a potential selectivity-conferring residue. In the binary complexes of LmNMT and HsNMT, the tyrosine appears as two conformers with inward and outward side-chain orientations. For HsNMT the inwardly oriented conformation appears to be preferred, whereas for LmNMT the outward orientation predominates (Fig. 5 ▶ *b*). This may be significant because the LmNMT inhibitors are juxtaposed differently with respect to this tyrosine residue. The ligands with the higher selectivity towards the *Leishmania* NMTs (compounds 7AH, A6K and CWZ) bind to the enzyme so as to occlude the inwardly oriented conformation of the side chain of the tyrosine residue (Fig. 5 ▶ *c*) and would clash with the preferred conformer of HsNMT Tyr296. In contrast, the less selective ligands 646 and 6KV allow the inward orientation of this aromatic side chain. This would introduce selectivity if the Δ*G* value for the inward to outward transition in the Tyr conformer is more positive for Tyr296 in HsNMT than it is for Tyr217 in the *Leishmania* NMTs. The potential role of the conformation of the corresponding tyrosine residue (211) in determining the selectivity of benzofuran-based inhibitors for *P. falciparum* NMT over HsNMT has been discussed previously (Yu *et al.*, 2012 ▶).

## 4. Summary and perspectives   

The structures presented here reveal the mode of binding of a set of four *Leishmania* NMT inhibitors emerging from a high-throughput screen. The three compounds with higher ligand efficiency each interact with the C-terminal carboxylate of the enzyme through a basic centre. Each inhibitor develops significant interactions with the aromatic side chains of Phe90, Tyr217 and Tyr345 as well as a set of interactions characteristic of each ligand. The next step is to use medicinal chemistry approaches to develop these hits into leads which inhibit in the nanomolar range. The higher ligand efficiency of the 6KV, 7AH and A6K compounds suggest that the prospects for success in developing higher affinity are good. The potent inhibitor 646 makes a polar interaction with Ser330 and such an interaction could be engineered into compounds from these three series, most notably 7AH which reaches up towards this residue. On the other hand, the CWZ molecule, which does form a polar contact with Ser330, could be adapted to form a direct interaction with the α-carboxylate. The ligands approach the Leu421 carboxylate from different directions and moreover they use different basic/hydrogen-bond donor groups to form interactions with it, suggesting that there is scope for the energetic contribution of this interaction to be augmented.

The structures of the bound inhibitors, which explore different aspects of the peptide-binding pocket in NMT, indicate that chimaeric molecules may be an effective route to explore possibilities for increased binding. For example, the close superposition of the second (chloro)phenyl ring of compound A6K and the six-membered ring of the indole of 7AH in their complexes with LmNMT suggests that hybrid molecules could be developed combining the higher affinity binding determinants of each molecule. Another possibility would be to exploit the close overlap of the core elements of 646 and 6KV by grafting the pyrazole element of the latter onto the former. A third possibility arises from the binding of two molecules of 6KV to adjacent sites in the inhibitor pocket, which suggests that a higher affinity covalently linked dimer of this molecule could be developed. Finally, we note that the open character of the active site gives scope for finding additional binding compounds through further screening.

## Supplementary Material

PDB reference: LmNMT–MyrCoA, 4cgp

PDB reference: LmNMT–MyrCoA–6KV, 4cgo

PDB reference: LmNMT–MyrCoA–7AH, 4cgn

PDB reference: LmNMT–MyrCoA–A6K, 4cgl

PDB reference: LmNMT–MyrCoA–CWZ, 4cgm

Characterisation data for resynthesed compounds and three supplementary figures.. DOI: 10.1107/S2052252514013001/jt5003sup1.pdf

## Figures and Tables

**Table 1:** Drug-like lead compounds

				IC_50_ (µ*M*)		
Compound class and code†	Molecular structure	MW‡	LE§	LdNMT	LmNMT	HsNMT
Thienopyrimidine (6KV)	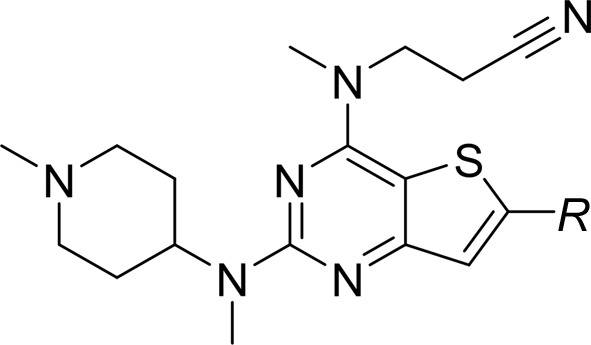	344	0.34	0.247 ± 0.008 (*n* = 6)	0.299 ± 0.089 (*n* = 4)	3.56 ± 0.49 (*n* = 6)
PF-00349412 (*R* = Me)						
IMP-0000083 (*R* = H)						
Piperidinylindole (7AH)	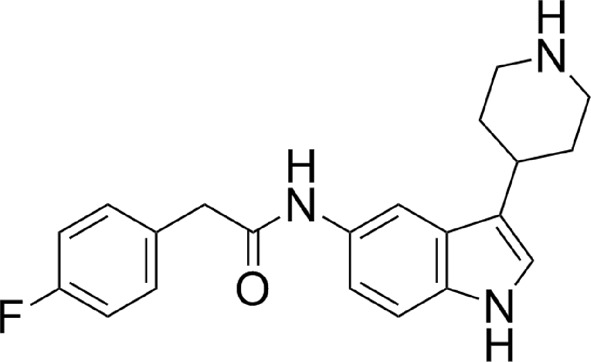	351	0.37	0.318 ± 0.101 (*n* = 2)	0.55 ± 0.07 (*n* = 2)	59.2 ± 17.1 (*n* = 2)
PF-03393842						
IMP-0000556						
Aminoacylpyrrolidine (A6K)	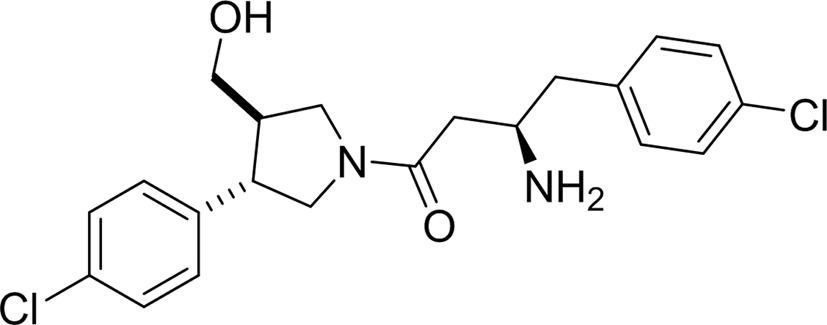	407	0.35	0.077 ± 0.007 (*n* = 4)	0.031 ± 0.004 (*n* = 2)	5.16 ± 0.83 (*n* = 2)
PF-03402623						
IMP-0000195						
Biphenyl derivative (CWZ)	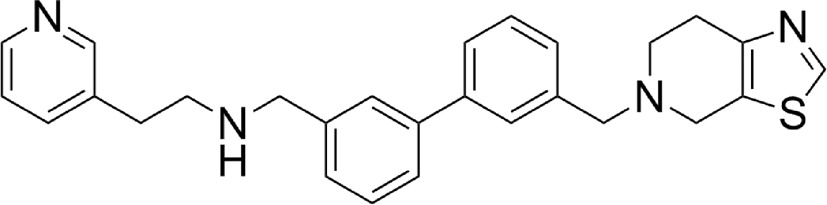	441	0.29	0.914 ± 0.089 (*n* = 2)	1.02 ± 0.10 (*n* = 2)	45.5 ± 6.3 (*n* = 2)
PF-00075634						
IMP-0000197						
DDD85646 (646)	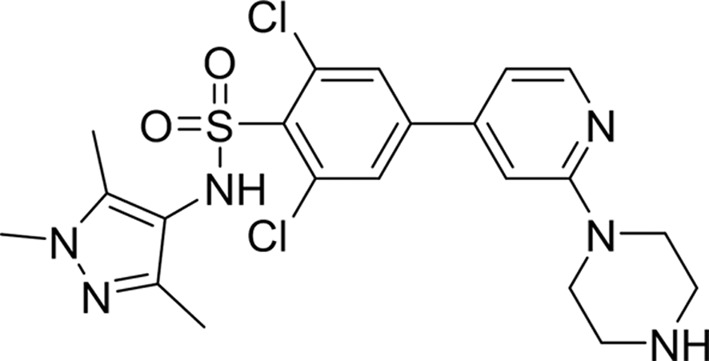	495	0.36	<0.006 (*n* = 4)	<0.006 (*n* = 2)	0.012 ± 0.002 (*n* = 6)
IMP-0000336						

† Protein Data Bank three-letter compound codes, PF code as designated by Bell *et al.* (2012 ▶) and IMP code designation for the resynthesized compound (Imperial College, London).

‡ Molecular weight (g mol^−1^).

§ Ligand efficiency LE = 1.4(−logLdNMTIC_50_)/*N*, where *N* is the number of non-H atoms (Hopkins *et al.*, 2014 ▶).

**Table 2:** X-ray diffraction data and refinement statistics Values in parentheses are for the highest resolution shell.

	LmNMT–MyrCoA	LmNMT–MyrCoA–6KV	LmNMT–MyrCoA–7AH	LmNMT–MyrCoA–A6K	LmNMT–MyrCoA–CWZ
PDB code	4cgp	4cgo	4cgm	4cgn	4cgl
Unit-cell parameters					
*a* (Å)	47.71	47.81	48.46	48.22	48.46
*b* (Å)	91.20	90.63	92.09	91.28	92.16
*c* (Å)	53.02	53.20	53.64	53.23	53.67
α = γ (°)	90.0	90.0	90.0	90.0	90.0
β (°)	111.7	111.9	113.7	113.3	113.7
Space group	*P*2_1_	*P*2_1_	*P*2_1_	*P*2_1_	*P*2_1_
Data collection					
Beamline	DLS I04	DLS I04-1	DLS I04	DLS I04	DLS I04-1
Wavelength (Å)	0.9795	0.9173	0.9795	0.9795	0.9173
Detector type	ADSC Q315 CCD	CMOS Pilatus 2M	ADSC Q315 CCD	ADSC Q315 CCD	CMOS Pilatus 2M
No. of images	400	1100	360	400	1100
Oscillation (°)	0.5	0.2	0.5	0.5	0.2
Resolution (Å)	49–1.40 (1.42–1.40)	49–1.30 (1.32–1.30)	31–1.70 (1.73–1.70)	49–1.69 (1.72–1.69)	46–1.48 (1.51–1.48)
*R*_merge_ † (%)	6.2 (31.1)	4.6 (62.5)	5.7 (62.6)	7.5 (76.0)	6.2 (96.3)
〈*I*/σ(*I*)〉	11.4 (2.8)	14.7 (1.9)	10.6 (1.8)	10.2 (1.6)	10.8 (1.6)
Completeness (%)	97.5 (76.5)	96.2 (70.0)	100 (100)	99.7 (99.8)	96.0 (96.0)
Multiplicity	4.0 (2.7)	4.1 (3.3)	3.8 (3.8)	4.1 (4.0)	4.2 (4.2)
Refinement					
No. of unique reflections	80714	99146	47383	47217	68755
*R*_work_/*R*_free_ ‡ (%)	15.9/19.6	16.2/19.6	18.5/24.6	17.6/22.4	20.0/24.2
No. of atoms					
Total	4326	4243	4093	3952	3940
Protein	3606	3476	3478	3466	3451
Ligand	n/a	48	32	26	27
Cofactor	63	63	63	63	63
Water	656	575	518	396	398
*B* factors (Å^2^)					
All atoms	12.8	16.5	25.2	19.5	22.7
Protein	10.7	14.6	23.7	18.5	21.8
Ligand	n/a	14.7/30.1§	13.6	15.0	12.4
Cofactor	7.4	10.5	17.3	12.8	14.3
Water	24.7	28.1	35.1	27.7	30.5
R.m.s. deviations¶					
Bond lengths (Å)	0.029	0.027	0.022	0.021	0.024
Bond angles (°)	2.670	2.640	2.184	2.120	2.330

† *R*_merge_ = 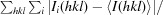 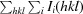, where *I_i_*(*hkl*) is the *i*th observation of reflection *hkl* and 〈*I*(*hkl*)〉 is the weighted average intensity for all observations *i* of reflection *hkl*.

‡ *R*_cryst_ = 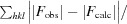 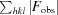, where *F*_obs_ and *F*_calc_ are the observed and calculated structure-factor amplitudes, respectively. *R*_free_ is the *R*_cryst_ calculated with 5% of the reflections omitted from refinement.

§ Values for high-affinity (P) and low-affinity (D) binding sites.

¶ Root-mean-square deviation of bond lengths or bond angles from ideal geometry.

**Table 3:** Residue surface area buried upon inhibitor binding The surface area of each residue buried by the binding of the inhibitor was determined using *PISA* (Krissinel & Henrick, 2007 ▶). Interactions involving a direct hydrogen-bonding, ion–dipole or ion–ion interaction with the ligand are denoted in bold.

Protein residue		Surface area buried by inhibitor (Å^2^)					
LmNMT/LdNMT	HsNMT	6KV (P)	6KV (D)	7AH	A6K	CWZ	646
Tyr80		10		6	13	11	9
Val81		34	16	7	32	32	45
Glu82			6	4		2	3
Asp83			38	18		20	21
Phe88			31	11		14	19
Phe90		34	25	50	34	52	47
Tyr92		9		7	9	11	6
Asn167		14		6	**18**	5	14
Thr203		18		11	**18**	11	19
Ala204		8		1	6	6	5
Gly205		27			11	12	30
Tyr217		44		34	49	58	43
Phe218	Trp				5	2	
His219		16	15	18	2	7	17
Phe232		4	51	13		20	21
Tyr326				6	**9**	7	
Ser330			8	6		**5**	**10**
Leu341			8	7		7	6
Ala343			5				2
Tyr345		**16**	6	**34**	24	31	8
Asn376		4	5	**11**	14	19	11
Met377	Ala			7	7	9	
Val378	Leu			13	17	12	
Gly395			5			3	
Asp396		1	42			4	3
Gly397		16					23
His398	Asn	5					1
Leu399		17		23	23	20	14
Met420	Leu	9		13	18	10	7
Leu421	Gln	**16**		**18**	**12**	8	**13**
MyrCoA		49		1	51	35	44
6KV(P)			84				
6KV(D)		84					
