# Supplementary material for: Diverse modes of binding in structures of Leishmania major N-myristoyltransferase with selective inhibitors
Source: IUCrJ. 2014 Jun 17;1(Pt 4):250–60. doi: 10.1107/S2052252514013001 (PMC4107925; doi:10.1107/S2052252514013001)
Supplement: Supplementary file 1 [file m-01-00250-sup1.pdf]

IUCrJ

Volume 1 (2014)

Supporting information for article:

Diverse modes of binding in structures of *Leishmania major N*-myristoyltransferase with selective inhibitors

James A. Brannigan, Shirley M. Roberts, Andrew S. Bell, Jennie A. Hutton, Michael R. Hodgkinson, Edward W. Tate, Robin J. Leatherbarrow, Deborah F. Smith and Anthony J. Wilkinson

S1. Characterisation data for the resynthesised compounds

The purity of synthesised compounds was verified by reverse phase LC-MS on a Waters 2767 system equipped with a photodiode array and an ESI mass spectrometer using a XBridge C18 (5 µm, 4.6 mm × 100 mm) column, equipped with an XBridge C18 guard column (5 µm, 4.6 mm × 20 mm). The mobile phase consisted of H_2O + 0.1 % formic acid (solvent A) and MeOH + 0.1% formic acid (solvent B). The following elution method was used: 0-10 min 5-98% B, 10-12 min 98% B, 12-13 min 98 to 5% B, 13-18 min 5% B at a flow rate of 1.2 ml/min. Purity of tested compounds was ≥ 95%, and the retention time (Rt) is reported in minutes. ^{1}H and ^{13}C NMR spectra were recorded on 500 MHz and 126 MHz respectively Bruker AV instruments at room temperature and were referenced to residual solvent signals. Data are presented as follows: chemical shift in ppm, multiplicity (br = broad, app = apparent, s = singlet, d = doublet, t = triplet, q = quartet, m = multiplet), integration and coupling constants in Hz. Mass spectra were obtained from the Mass Spectrometry Service of Department of Chemistry, Imperial College London. Full synthetic details will be published elsewhere in due course.

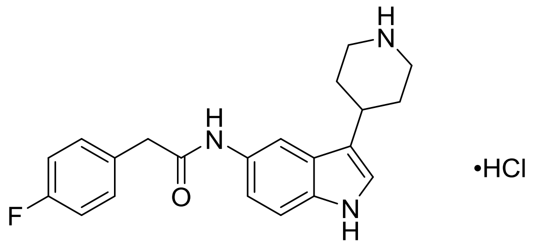

7AH

^{1}H NMR (CD_3OD) δ 8.01 (d, 1H, J = 2.0 Hz), 7.45 – 7.40 (m, 2H), 7.33 (d, 1H, J = 8.0 Hz), 7.12 – 7.05 (m, 4H), 3.71 (s, 2H), 3.52 – 3.49 (m, 2H), 3.21 – 3.17 (m, 3H), 2.31 – 2.26 (m, 2H), 2.00 – 1.95 (m, 2H); m/z: 352 ($[M+H]^+$); HRMS found 352.1823, $C_{21}H_{23}N_3OF$ requires 352.1825; LCMS Rt = 10.28 min.

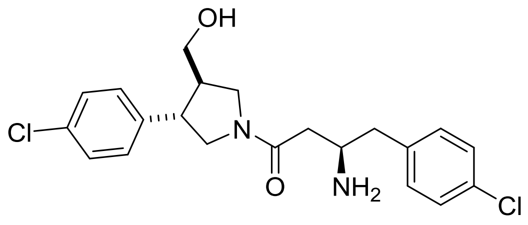

A6K

^{1}H NMR (400 MHz, MeOD) δ 7.43 – 7.24 (m, 8H), 4.06 – 3.76 (m, 2H), 3.72 (s, 1H), 3.61 – 3.55 (m, 1H), 3.52 – 3.37 (m, 3H), 3.29 – 3.14 (m, 1H), 2.99 – 2.85 (m, 2H), 2.72 – 2.39 (m,

3H); m/z 407 ([M+H]$^+$), HRMS found 407.1313, $C_{21}H_{25}N_2O_2Cl_2$ requires 407.1293; LCMS Rt = 12.44 min.

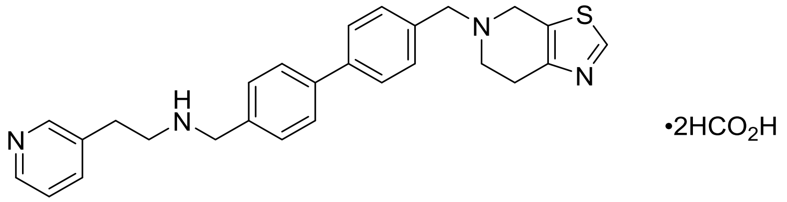

CWZ

^{1}H NMR (400 MHz, MeOD) δ 8.86 (s, 1H), 8.53 – 8.41 (m, 2H), 8.28 (s, 2H), 7.83 – 7.72 (m, 4H), 7.63 (dt, J = 7.7, 1.5 Hz, 1H), 7.56 (t, J = 7.7 Hz, 1H), 7.52 – 7.46 (m, 2H), 7.46 – 7.41 (m, 2H), 4.32 (s, 2H), 3.91 (s, 2H), 3.85 (s, 2H), 3.39 – 3.33 (m, 2H), 3.12 – 3.05 (m, 2H), 3.05 – 2.99 (m, 2H), 2.99 – 2.91 (m, 2H); m/z 441 ([M+H]$^+$), HRMS found 441.2119, $C_{27}H_{29}N_4OS$ requires 441.2113; LCMS Rt = 7.26 min.

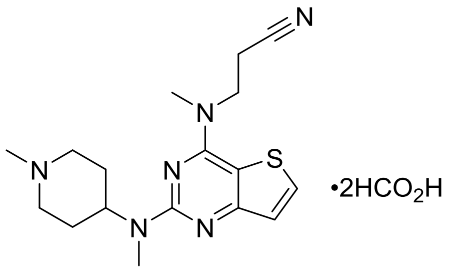

6KV

^{1}H NMR (400 MHz, MeOD) δ 8.41 (s, 2H), 7.96 (d, J = 5.6 Hz, 1H), 7.23 (d, J = 5.5 Hz, 1H), 5.00 (m, 1H), 4.11 (t, J = 6.6 Hz, 2H), 3.68 – 3.61 (m, 2H), 3.59 (s, 3H), 3.23 (td, J = 12.8, 3.0 Hz, 2H), 3.10 (s, 3H), 2.93 (t, J = 6.7 Hz, 2H), 2.91 (s, 3H), 2.22 (qd, J = 13.1, 3.7 Hz, 2H), 2.09 – 1.93 (m, 2H); m/z 345 ([M+H]$^+$), HRMS found 345.1875, $C_{17}H_{24}N_6S$ requires 345.1861; LCMS Rt = 5.9 min.

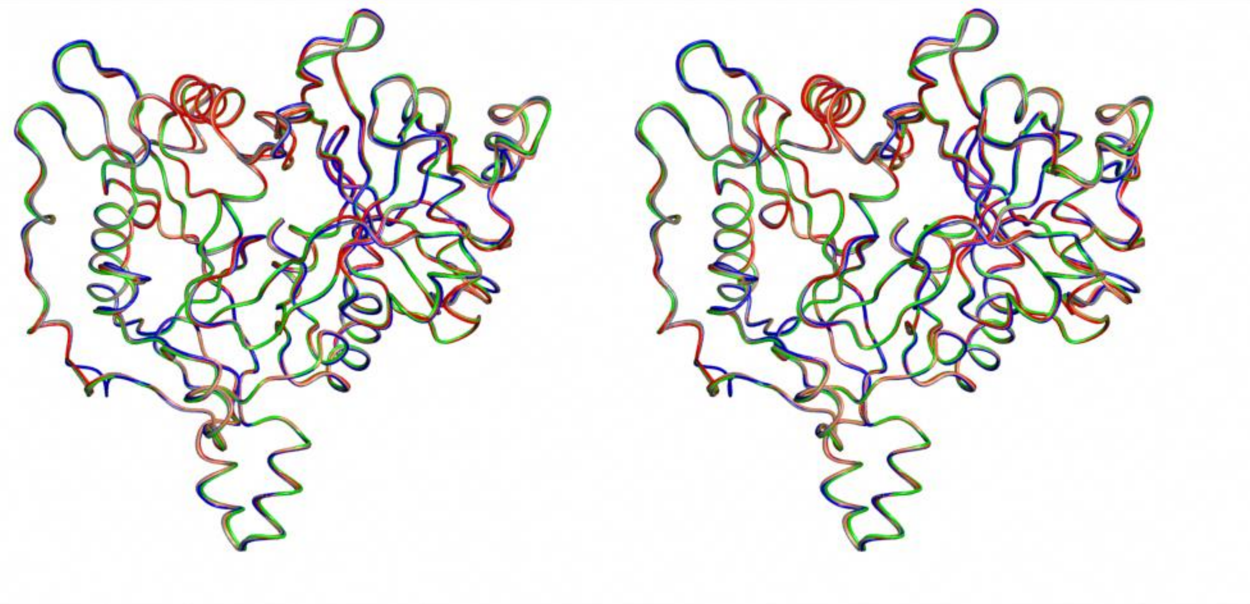

A

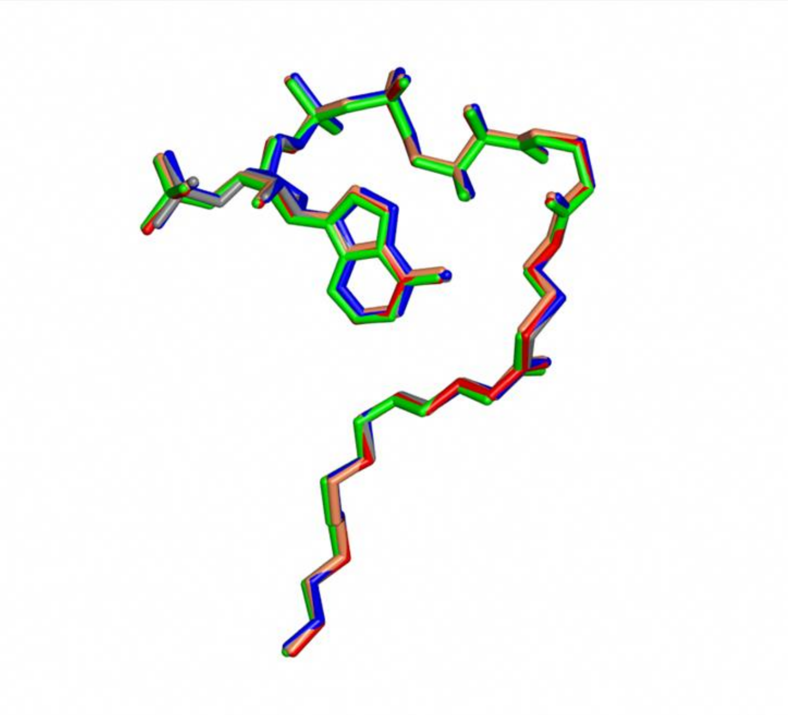

B

Figure S1 . (A) Stereo superposition of the structure of binary complex of LmNMT with MyrCoA (blue) with the ternary complexes of LmNMT-MyrCoA with inhibitors 6KV (grey), 7AH (coral), A6K (green) and CWZ (red). The molecules are shown as worm tracings following superposition using the secondary structure mapping routines implemented in CCP4mg. (B) Invariance of MyrCoA binding across the structures. The MyrCoA ligands from the five structures (coloured as above) are shown after superposition of the models as described above.

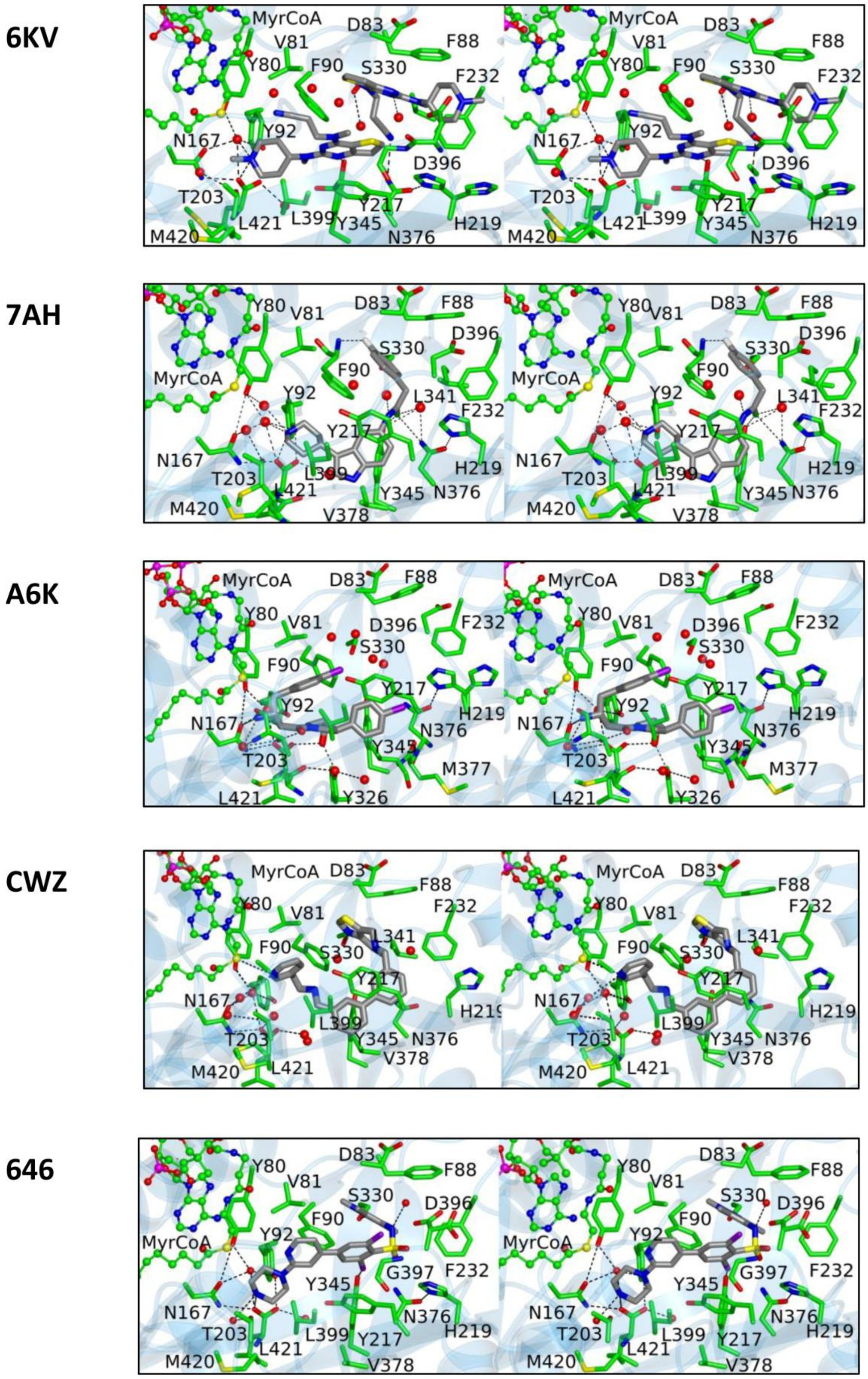

Figure S2 Stereo-views of inhibitor binding. Atom colours; carbon (green for protein, grey for ligand), oxygen (red), nitrogen (blue), sulphur (yellow), fluorine (turquoise) and chlorine (white).

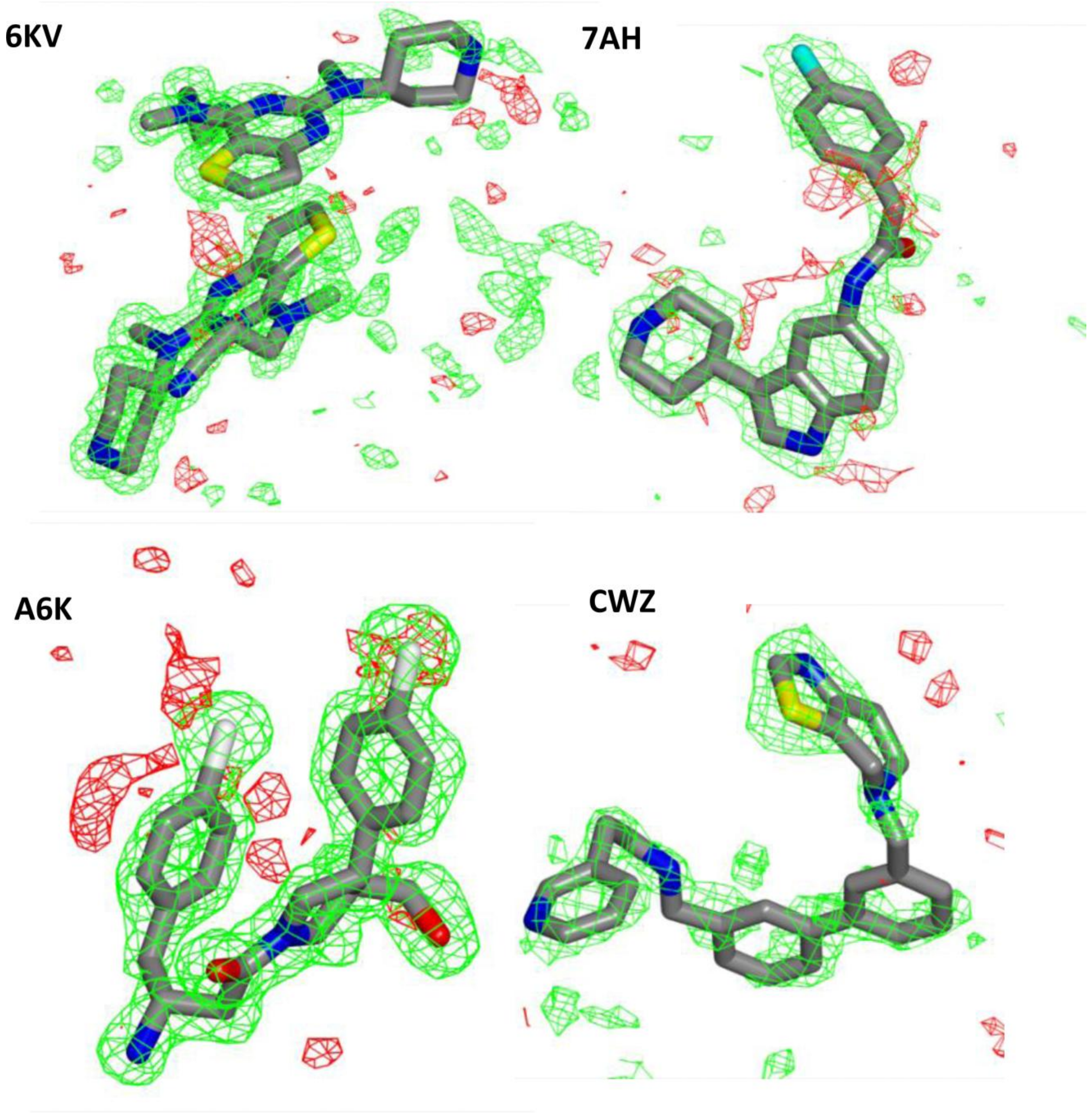

Figure S3 'Omit' maps. Initial electron density maps calculated at preliminary stages of refinement (in the absence of ligand) reveal regions of positive (red) and negative (green) density in the difference map (mF_O-dF_C). The ligands are shown in cylinder representation, coloured by atom; carbon (gray), oxygen (red), nitrogen (blue), sulphur (yellow), fluorine (turquoise) and chlorine (white).
